# Construction and validation of a machine learning-based nomogram to predict the prognosis of HBV associated hepatocellular carcinoma patients with high levels of hepatitis B surface antigen in primary local treatment: a multicenter study

**DOI:** 10.3389/fimmu.2024.1357496

**Published:** 2024-03-27

**Authors:** Yiqi Xiong, Wenying Qiao, Qi Wang, Kang Li, Ronghua Jin, Yonghong Zhang

**Affiliations:** ^1^ Interventional Therapy Center for Oncology, Beijing You’an Hospital, Capital Medical University, Beijing, China; ^2^ Research Center for Biomedical Resources, Beijing You’an Hospital Capital Medical University, Beijing, China; ^3^ National Center for Infectious Diseases, Beijing Ditan Hospital, Capital Medical University, Beijing, China; ^4^ Interventional Radiology Department, Beijing Friendship Hospital, Capital Medical University, Beijing, China; ^5^ Research Center for Biomedical Resources, Beijing You’an Hospital, Capital Medical University, Beijing, China

**Keywords:** hepatocellular carcinoma, hepatitis B surface antigen (HBsAg), TACE, ablation, nomogram, recurrence

## Abstract

**Background:**

Hepatitis B surface antigen (HBsAg) clearance is associated with improved long-term outcomes and reduced risk of complications. The aim of our study was to identify the effects of levels of HBsAg in HCC patients undergoing TACE and sequential ablation. In addition, we created a nomogram to predict the prognosis of HCC patients with high levels of HBsAg (≥1000U/L) after local treatment.

**Method:**

This study retrospectively evaluated 1008 HBV-HCC patients who underwent TACE combined with ablation at Beijing Youan Hospital and Beijing Ditan Hospital from January 2014 to December 2021, including 334 patients with low HBsAg levels and 674 patients with high HBsAg levels. The high HBsAg group was divided into the training cohort (N=385), internal validation cohort (N=168), and external validation cohort (N=121). The clinical and pathological features of patients were collected, and independent risk factors were identified using Lasso-Cox regression analysis for developing a nomogram. The performance of the nomogram was evaluated by C-index, receiver operating characteristic (ROC) curves, calibration curves, and decision curve analysis (DCA) curves in the training and validation cohorts. Patients were classified into high-risk and low-risk groups based on the risk scores of the nomogram.

**Result:**

After PSM, mRFS was 28.4 months (22.1-34.7 months) and 21.9 months (18.5-25.4 months) in the low HBsAg level and high HBsAg level groups (P<0.001). The content of the nomogram includes age, BCLC stage, tumor size, globulin, GGT, and bile acids. The C-index (0.682, 0.666, and 0.740) and 1-, 3-, and 5-year AUCs of the training, internal validation, and external validation cohorts proved good discrimination of the nomogram. Calibration curves and DCA curves suggested accuracy and net clinical benefit rates. The nomogram enabled to classification of patients with high HBsAg levels into low-risk and high-risk groups according to the risk of recurrence. There was a statistically significant difference in RFS between the two groups in the training, internal validation, and external validation cohorts (P<0.001).

**Conclusion:**

High levels of HBsAg were associated with tumor progression. The nomogram developed and validated in the study had good predictive ability for patients with high HBsAg levels.

## 1 Introduction

Primary liver cancer is the sixth most common cancer and the second leading cause of cancer death worldwide, which poses a huge economic and disease burden worldwide due to its high morbidity and mortality rates (1, 2). China is the country with the highest hepatocellular carcinoma (HCC) occurrence and the overall incidence of HCC is expected to continue to climb (3). HCC occurs most often in the setting of chronic liver inflammation and is mainly induced by hepatitis B virus (HBV) infection (4), which is a key risk factor for liver cirrhosis and HCC, capable of increasing the risk of HCC approximately 20-fold (5–7). For early HCC, surgical resection, liver transplantation, and ablation are recommended treatments. Studies have shown that ablation has similar five-survival rates compared to surgical treatment, and fewer complications than surgery (8, 9). However, the recurrence rate after ablation remains high, with a five-year recurrence rate of 50-70% (10). Transcatheter arterial chemoembolization (TACE) is the only guideline-recommended global standard of care for intermediate-stage HCC, and the median progression-free survival time (mPFS) is only 5 months (11). Therefore, diagnosis and treatment of HCC is an increasingly important public health problem.

The first serologic marker of HBV infection is Hepatitis B surface antigen (HBsAg), which can be detected from 2 to 12 weeks after infection with HBV (12). HBsAg clearance, which is currently regarded as the functional cure of chronic hepatitis (CHB), is associated with improved long-term outcomes and reduced risk of complications (13, 14). The decline in HBsAg during antiviral therapy is relatively slow, and the seroclearance rate is faster at low serum HBsAg expression (<1000U/L) (15, 16). Previous studies revealed that high serum levels of HBsAg increase the risk of developing HCC and have a worse prognosis for patients who have already developed HCC (17). Nevertheless, the prognostic impact of serum HBsAg levels in patients after TACE sequential ablation therapy needs to be further confirmed.

HBV-HCC prognosis is linked to several factors, including tumor burden, AFP, disease stage, ALBI, and NLR (18, 19), and there are also nomograms about HBV-HCC (20–22). However, no nomogram for HCC patients with high HBsAg expression after local treatment has been available to our knowledge. We compared the effects of high levels of HBsAg (≥1000U/L) and low levels of HBsAg (<1000U/L) in HCC patients undergoing TACE and sequential ablation and utilized propensity score matching to minimize selection bias. In addition, we created a nomogram to predict the prognosis of HCC patients with high levels of HBsAg after local treatment to more accurately guide the clinical decision.

## 2 Materials and methods

### 2.1 Patient selection

**Figure 1:**
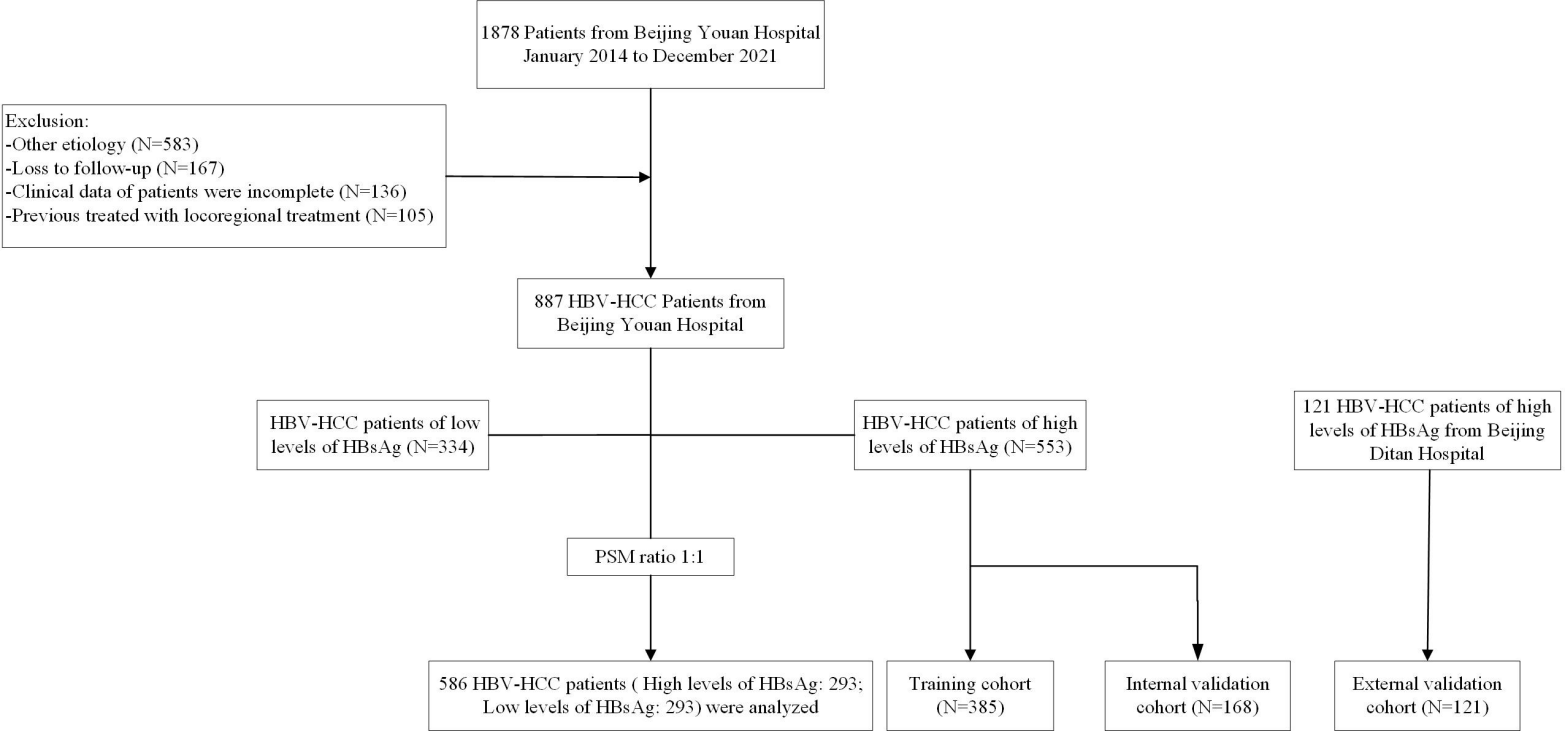
Screening flow chart of enrolled patients.

This study retrospectively evaluated 1008 HBV-HCC patients who underwent TACE combined with ablation at Beijing Youan Hospital and Beijing Ditan Hospital from January 2014 to December 2021. The diagnosis of HCC was based on the guideline of the America Association for the Study of Liver Diseases (ASSLD) (1, 23). The patients at Youan Hospital consisted of 553 patients with a high level of HBsAg and 334 patients with a low level of HBsAg. In order to build a reliable model, the patients from Youan Hospital were divided into the training cohort (N=385) and the validation cohort (N=168). Furthermore, 121 patients from Ditan Hospital were used as an independent external verification cohort to verify the external applicability of the nomogram. The inclusion criteria of patients were as follows (1): Aged 18-80 years (2). received TACE combined ablation (3). Child-Pugh classification was class A or B (4). all patients had not received any other therapeutics before ablation. Exclusion criteria were listed as follows (1): with second primary malignant tumors (2). clinical follow-up data incomplete (3). advanced HCC. (**Figure 1**).

The study was approved by the Medical Ethics Committee of Youan Hospital and Ditan Hospital and was performed in compliance with the standards of the Helsinki Declaration. The requirement for informed consent was waived because the study was deemed to pose no additional risk to patients and the data were deidentified.

### 2.2 Clinicopathologic characteristics

The demographic, clinical, and histopathologic data of patients were collected. Demographics included age, sex, drinking history, smoking history, hypertension and diabetes. Clinical and pathological data was composed of tumor size, tumor number, alpha-fetoprotein (AFP), aspartate aminotransferase (AST), alanine aminotransferase (ALT), gamma glutamyl transferase (GGT), albumin (ALB), neutrophil-to-lymphocyte ratio (NLR), platelet-to-lymphocyte ratio (PLR), and gamma glutamyl transferase to lymphocyte ratio (GLR).

### 2.3 Treatment received

#### 2.3.1 TACE procedure

TACE was conducted by experienced interventional radiologists. Under local anesthesia, percutaneous right femoral artery puncture with a modified Seldinger technique was performed. Angiography was conducted by the 5-F (Terumo, Tokyo, Japan) catheter to identify arterial supply to tumors and to assess the patency of the portal vein. When applicable, a microcatheter was inserted into the blood-supply artery of the carcinoma to inject a mixture of doxorubicin (Pfizer Inc., New York, NY, USA) and lipiodol (Guerbet, Villepinte, France), followed by embolization using embolic materials, such as gelfoam or polyvinyl alcohol particles. The blood flow was monitored until complete vessel occlusion was observed. TACE was repeated thereafter if the lesion is not completely necrotic and the active portion exceeds 50% of the baseline value.

#### 2.3.2 Ablation procedure

Performed under the guidance of computed tomography (CT) and magnetic resonance imaging (MRI) by a qualified interventionalist. The size of the tumor decided the number of electrodes. Routine disinfection and intravenous anesthesia were applied around the puncture points. During RFA, after measuring the baseline impedance, the power was gradually increased from 80w to 200w to reach the maximum impedance. The electrode tip temperature was kept below 20°C by the pump injected cold brine into the electrode chamber. Moreover, to achieve complete ablation, the safe margin for complete ablation of the tumor was 0.5cm. After ablation, the needle track was ablated to prevent postoperative bleeding and tumor implantation along the needle track. Arteriography-enhanced CT was performed immediately after treatment to evaluate the success of the procedure and its complications.

### 2.3 Follow-up

All patients underwent regular follow-ups at the outpatient clinics. Tumor responses were evaluated at approximately 4-6 weeks after ablation by using CT or MRI. For the follow-up protocol, patients were examined every 3 months during the first year and every 6 months thereafter. The contents of the follow-up included blood tests, liver function, and imaging examination to detect tumor recurrence. The study endpoint was recurrence-free survival (RFS), defined as the time from ablation to the first recurrence.

### 2.4 Statistical analysis

Differences between the groups were compared through the t-test, chi-square test, Mann-Whitney U test, and Kruskal-Wallis test, with the purpose of providing median or counts and percentages to summarize baseline variables. Survival and recurrence were calculated using the Kaplan-Meier method, and the log-rank test was used for comparison. Lasso regression was performed for risk factor selection and identified independent risk factors for tumor recurrence were used in Multivariate Cox regression analysis. A nomogram based on independent risk factors to predict recurrence. Subsequently, the performance of the nomogram was validated in the internal validation and the external validation cohort. According to the nomogram scores, the patients were classified as low-risk and high-risk groups, and their recurrence rates were predicted. The receiver operating characteristic (ROC) curves were plotted and the area under the curves (AUCs) was calculated to evaluate prognostic value. Calibration curves and the Hosmer-Lemeshow test were conducted to assess the predictive ability of the nomogram. To estimate the clinical utility of the nomogram, decision curve analysis (DCA) was conducted by calculating the net benefits for a range of threshold probabilities.

To reduce the potential selection bias, 1:1 propensity score matching (PSM) was conducted, with a matching tolerance was 0.1. Matches were made in baseline variables that were previously considered clinically relevant in the literature, comprising age, sex, Child-pugh classification, BCLC stage, tumor size, tumor number, ALT, AST, and AFP.

All data were analyzed with SPSS (version 26.0, IBM, Armonk, NY, USA) and R software (version 4.1.3) in this study, and a P-value less than 0.05 was considered statistically significant (two-tailed tests).

## 3 Result

A total of 1008 HBV-HCC patients from Beijing Youan Hospital and Beijing Ditan Hospital were screened between January 1, 2014, to December 31, 2021, including 334 patients with low HBsAg levels and 674 patients with high HBsAg levels. After PSM, 293 patients were included in each group (**Figure 1**). The high levels of HBsAg groups were divided into the training cohort (N=385), internal validation cohort (N=168), and external validation cohort (N=121). The last follow-up until July 1, 2023, and the median follow-up time was 4.05 years (25~75th percentiles, 2.68~7.05 years).

Before PSM, baseline data showed that compared to the low HBsAg level group, the high HBsAg level group had a younger age (55.9 ± 9.03 VS. 58.3 ± 8.34, P<0.001), lower levels of TBIL (18.64 ± 9.42 VS. 20.84 ± 11.37, P=0.002), and shorter TT (15.77 ± 2.12VS. 15.95 ± 2.39, P=0.008). After PSM, all demographic and clinicopathologic data were well balanced between the two groups (**Table 1**).

**Table 1:** Demographics and clinical characteristics before and after PSM.

	Before PSM			After PSM		
	Low HBsAg level (N=334)	High HBsAg level (N=553)	P value	Low HBsAg level (N=293)	High HBsAg level (N=293)	P value
Age Sex male female	58.3 ± 8.34 271 (81.1%) 63 (18.9%)	55.9 ± 9.03 439 (79.5%) 114 (20.5%)	<0.001 0.614	58.4 ± 8.40 233 (79.5%) 60 (20.5%)	58.6 ± 7.71 235 (80.2%) 58 (19.8%)	0.728 0.918
Diabetes Yes No	252 (75.4%) 82 (24.6%)	479 (75.9%) 74 (24.1%)	0.429	220 (75.1%) 73 (24.9%)	236 (80.5%) 57 (19.5%)	0.136
Child-Pugh class A B Cirrhosis Yes No BCLC stage 0 A B T.N Single Multiple T.S <30mm ≥30mm WBC (10^9/L) NLR MLR Hb (g/L) PLR TBIL (umol/L) GGT (U/L) GLR Fib (g/L) TT (s) Alb (g/L) Palb (g/L) ALT (U/L) AST (U/L) AFP (umol/L)	246 (73.7%) 88 (26.3%) 43 (12.9%) 291 (87.1%) 97 (29.0%) 187 (56.0%) 50 (15.0%) 237 (71.0%) 97 (29.0%) 215 (64.4%) 119 (35.6%) 5.06±2.17 3.17±2.62 0.38±0.23 128±20 111.61±61.43 20.84±11.37 67.48±64.43 68.06±90.12 2.81±1.02 15.95±2.39 37.19±4.78 139.09±60.38 29.28±16.95 31.51±14.09 382.96±1930.96	427 (77.3%) 126 (22.7%) 72 (13.1%) 481 (86.9%) 174 (31.4%) 291 (52.6%) 88 (16.0%) 378 (68.3%) 175 (31.7%) 357 (64.5%) 196 (35.5%) 5.22±2.16 3.28±2.84 0.37±0.21 131±19 108.78±54.90 18.64±9.42 68.15±58.89 69.12±84.74 2.84±0.88 15.77±2.12 37.21±4.88 139.32±58.59 32.55±19.96 32.27±15.66 341.67±1871.85	0.244 0.989 0.604 0.436 1 0.976 0.850 0.342 0.073 0.283 0.002 0.617 0.928 0.064 0.008 0.914 0.653 0.111 0.247 0.495	216 (73.7%) 77 (26.3%) 38 (13.0%) 255 (87.0%) 86 (29.4%) 168 (57.3%) 39 (13.3%) 213 (72.7%) 80 (27.3%) 192 (65.5%) 101 (34.5%) 5.00 ± 2.10 3.21 ± 2.67 0.38 ± 0.234 129 ± 20.4 113 ± 62.6 21.2 ± 11.6 65.6 ± 58.7 67.5 ± 90.8 2.78 ± 0.99 16.0 ± 2.38 37.3 ± 4.85 140 ± 60.7 29.4 ± 17.0 31.6 ± 13.9 337 ± 1800	216 (73.7%) 77 (26.3%) 42 (14.3%) 251 (85.7%) 85 (29.0%) 156 (53.2%) 52 (17.7%) 209 (71.3%) 84 (28.7%) 187 (63.8%) 106 (36.2%) 5.22 ± 2.22 3.43 ± 2.90 0.39 ± 0.216 131 ± 19.4 108 ± 51.7 19.4 ± 9.26 63.5 ± 53.7 64.0 ± 60.7 2.88 ± 0.92 15.8 ± 2.12 36.8 ± 4.94 131 ± 56.8 31.2 ± 19.6 32.4 ± 15.4 357 ± 1730	1 0.718 0.315 0.783 0.730 0.208 0.342 0.711 0.268 0.359 0.046 0.651 0.582 0.236 0.451 0.201 0.081 0.228 0.515 0.895

ALD, alcoholic liver cancer; BCLC, Barcelona Clinic Liver Cancer; T.N, tumor number; T.S, tumor size; WBC, leukocyte; Hb, hemoglobin; NLR, neutrophil-to-lymphocyte ratio; MLR, monocyte-to-lymphocyte ratio; PLR, platelet-to-lymphocyte ratio; ALT, alanine aminotransferase; AST, aspartate aminotransferase; TBIL, total bilirubin; ALB, albumin; GGT, gamma glutamyl transferase; GLR, gamma glutamyl transferase to lymphocyte ratio; Palb, prealbumin; Fib, fibrous protein; TT, thrombin time; AFP, alpha-fetoprotein.

The internal validation cohort and the external validation cohort had similar baseline characteristics to the training cohort. In the three cohorts, the majority of the patients were male (81.0%VS. 77.4%VS. 76.9%, p=0.466), and the average age was over 50 years(56.1 ± 9.10 VS. 56.6 ± 8.46 VS. 57.9 ± 8.57, P=0.466). Most patients were Child-Pugh A (76.9%VS. 80.4% VS., P=0.427), suggesting that the patients had good liver function. BCLC A had the highest percentage of patients (51.7% VS. 55.4% VS. 71.7%, P=0.182). Regarding tumor characteristics, most tumors were solitary (70.6% vs.71.4% VS. 67.8%, P=0.780) and tumor size was less than 3cm (70.6% vs. 69.0% VS. 67.8%, P=0.150) (**Table 2**).

**Table 2:** Demographics and clinical characteristics for training and validation sets.

	Training Cohort (N=385)	Internal Validation Cohort (N=168)	External Validation Cohort (N=121)	P-value
Age				
Mean ± SD	56.1 ± 9.10	56.6 ± 8.46	57.9 ± 8.57	0.150
**Sex**				0.466
Male	312 (81.0%)	130 (77.4%)	93 (76.9%)	
Female	73 (19.0%)	38 (22.6%)	28 (23.1%)	
**Hypertension**				0.617
No	292 (75.8%)	129 (76.8%)	97 (80.2%)	
Yes	93 (24.2%)	39 (23.2%)	24 (19.8%)	
**Diabetes**				0.559
No	310 (80.5%)	141 (83.9%)	101 (83.5%)	
Yes	75 (19.5%)	27 (16.1%)	20 (16.5%)	
**Antiviral**				0.137
No	158 (41.0%)	71 (42.3%)	62 (51.2%)	
Yes	227 (59.0%)	97 (57.7%)	59 (48.8%)	
**Smoking**				0.467
No	234 (60.8%)	94 (56.0%)	68 (56.2%)	
Yes	151 (39.2%)	74 (44.0%)	53 (43.8%)	
**Cirrhosis**				0.434
No	48 (12.5%)	27 (16.1%)	14 (11.6%)	
Yes	337 (87.5%)	141 (83.9%)	107 (88.4%)	
**ChildPugh**				0.182
A	296 (76.9%)	135 (80.4%)	86 (71.1%)	
B	89 (23.1%)	33 (19.6%)	35 (28.9%)	
**BCLC**				0.288
0	119 (30.9%)	56 (33.3%)	31 (25.6%)	
A	199 (51.7%)	93 (55.4%)	70 (57.9%)	
B	67 (17.4%)	19 (11.3%)	20 (16.5%)	
**T.N**				0.780
Single	272 (70.6%)	120 (71.4%)	82 (67.8%)	
Multiple	113 (29.4%)	48 (28.6%)	39 (32.2%)	
**T.S**				0.150
<3cm	234 (60.8%)	116 (69.0%)	73 (60.3%)	
≥3cm	151 (39.2%)	52 (31.0%)	39 (32.2%)	
**WBC(10^9/L)**				0.648
Mean ± SD	5.29 ± 2.22	5.15 ± 2.00	5.10 ± 2.17	
**NLR**				0.892
Mean ± SD	3.31 ± 2.93	3.19 ± 2.80	3.34 ± 3.10	
**MLR**				0.124
Mean ± SD	0.377 ± 0.216	0.355 ± 0.196	0.41 ± 0.24	
**PLR**				0.173
Mean ± SD	112 ± 58.8	102 ± 45.9	107 ± 56.2	
**ALT (U/L)**				0.101
Mean ± SD	33.1 ± 20.6	30.7 ± 17.7	29.3 ± 15.6	
**AST (U/L)**				0.180
Mean ± SD	33.0 ± 16.6	30.6 ± 13.0	33.5 ± 14.6	
**TBIL (umol/L)**				0.042
Mean ± SD	18.7 ± 9.59	18.6 ± 9.04	21.2 ± 12.2	
**DBIL (umol/L)**				0.777
Mean ± SD	6.41 ± 4.56	6.58 ± 4.40	6.19 ± 4.82	
**Total.alb (g/L)**				0.473
Mean ± SD	65.0 ± 8.52	64.6 ± 5.95	65.7 ± 6.74	
**Alb (g/L)**				0.887
Mean ± SD	37.1 ± 5.11	37.3 ± 4.56	37.0 ± 4.72	
**Globulin (g/L)**				0.041
Mean ± SD	28.4 ± 5.65	27.4 ± 4.99	28.9 ± 6.11	
**GGT (umol/L)**				0.484
Mean ± SD	69.8 ± 60.3	70.0 ± 67.6	77.7 ± 74.7	
**GLR**				0.362
Mean ± SD	69.8 ± 90.6	70.5 ± 77.9	83.6 ± 126	
**Bile.acid**				0.852
Mean ± SD	21.8 ± 30.2	20.5 ± 26.0	22.2 ± 23.9	
**Fib (g/L)**				0.348
Mean ± SD	2.85 ± 0.889	2.83 ± 0.896	2.71 ± 0.91	
**AFP (umol/L)**				0.707
Mean ± SD	412 ± 2240	266 ± 770	432 ± 2531	

BCLC, Barcelona Clinic Liver Cancer; ALD, alcoholic liver cancer; BCLC, Barcelona Clinic Liver Cancer; T.N, tumor number; T.S, tumor size; WBC, leukocyte; NLR, neutrophil-to-lymphocyte ratio; MLR, monocyte-to-lymphocyte ratio; PLR, platelet-to-lymphocyte ratio; ALT, alanine aminotransferase; AST, aspartate aminotransferase; TBIL, total bilirubin; DBIL, direct bilirubin; ALB, albumin; GGT, gamma glutamyl transferase; GLR, gamma glutamyl transferase to lymphocyte ratio; Fib, fibrous protein; AFP, alpha-fetoprotein.

### 3.1 Efficacy

**Figure 2:**
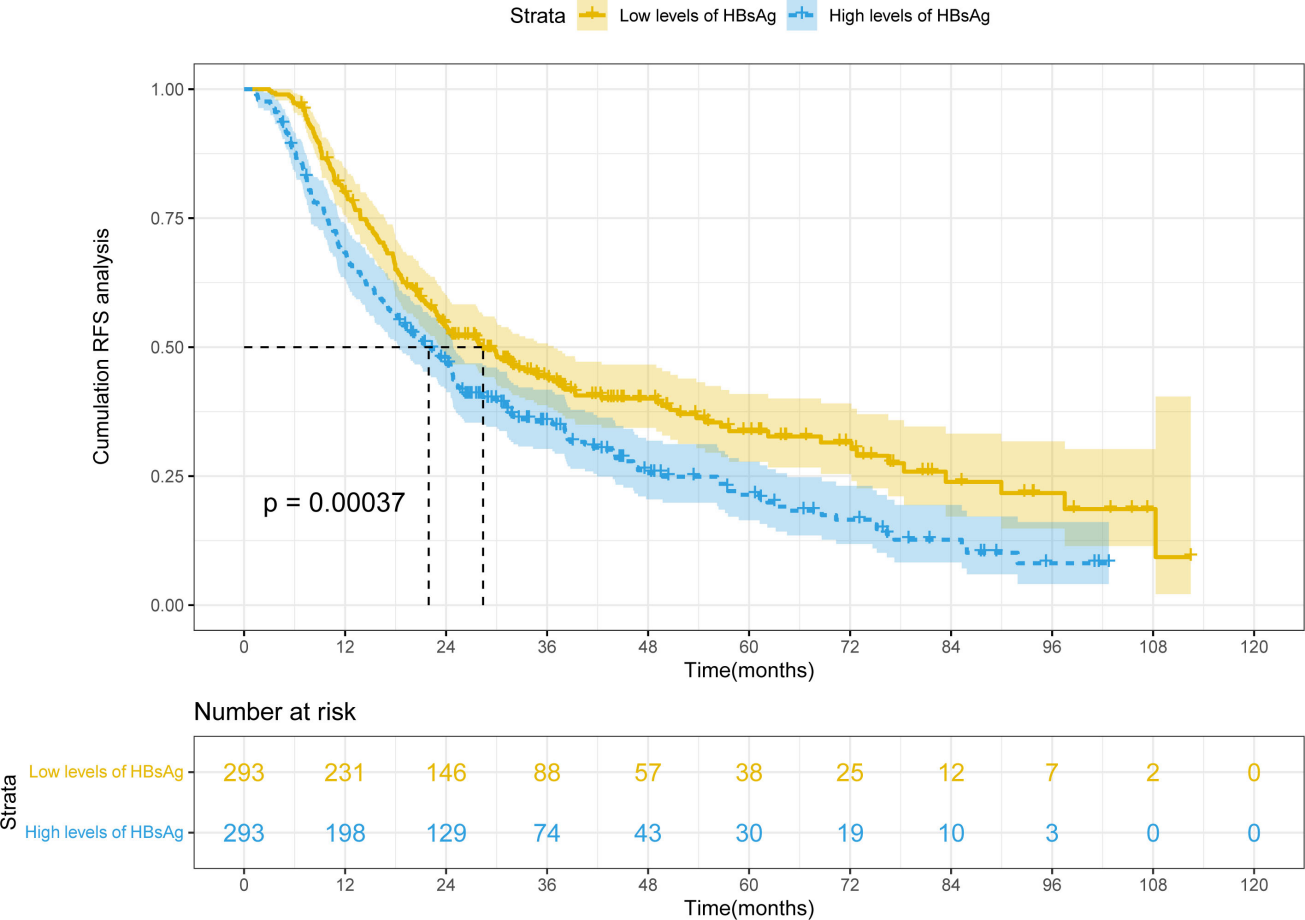
Kaplan-Meier plot of RFS for HBV-HCC after PSM.

After PSM, mRFS was 28.4 months (22.1-34.7 months) and 21.9 months (18.5-25.4 months) in the high HBsAg level and low HBsAg level groups, respectively (**Figure 2**). Because mRFS were significantly shorter in the high HBsAg level (P<0.001), a nomogram for predicting recurrence needs to be developed for the high HBsAg group in order to prompt clinical interventions.

### 3.2 The prediction model was built based on the Lasso-Cox regression

#### 3.2.1 Independent prognostic factors of RFS

**Figure 3:**
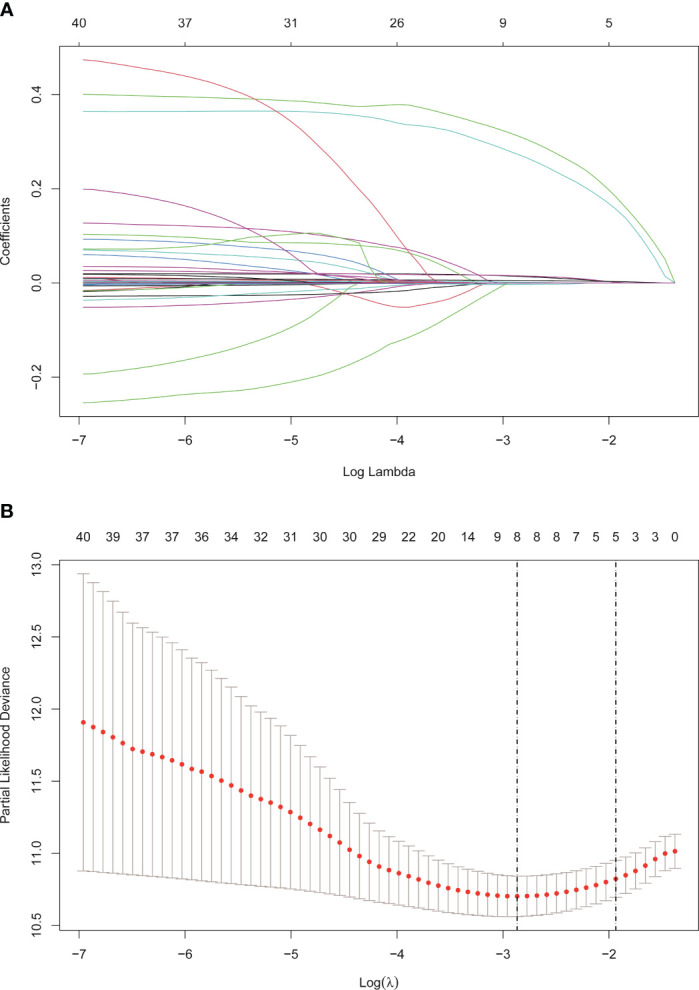
Screening of variables based on Lasso regression. **(A)** The variation characteristics of the coefficient of variables. **(B)** the selection process of the optimum value of the parameter λ in the Lasso regression model by cross-validation method.

The cohort in Beijing Youan Hospital was randomly split in a 7:3 ratio into the training (N=385) and internal validation (N=168) sets. The external validation cohort consisted of patients from Beijing Ditan Hospital. There were no statistical differences between the three groups (P<0.05), which showed that the data grouping was random and reasonable. Lasso regression was used to screen parameters, and the variation characteristics of the coefficient of these variables were shown in **Figure 3A**. The model exhibited outstanding performance and the least number of independent variables (**Figure 3B**). The screened variables included age, BCLC stage, tumor size, ALB, Palb, GLB, GGT, and bile acids. Variables screened based on Lasso regression were further subjected to multifactorial COX regression analysis to screen independent risk factors associated with recurrence (**Table 3**). The final results obtained were age (HR: 1.02, 95% CI: 1.01-1.04), BCLC stage (HR: 1.53, 95% CI: 1.22-1.91), tumor size (HR: 1.44, 95% CI: 1.06-1.94), globulin (HR: 1.02, 95% CI: 1-1.04), GGT (HR: 1.01, 95% CI: 1-1.01), and bile acids (HR: 1, 95% CI: 1-1.01).

**Table 3:** Cox proportional hazards regression to predict recurrence based on Lasso regression.

Variables	β	Z	HR (95%CI)	P value
Age	0.025	3.51	1.02 (1.01-1.04)	<0.001
BCLC	0.423	3.69	1.53 (1.22-1.91)	<0.001
T.S Alb Palb	0.364 -0.004 -0.001	2.37 -0.25 -0.895	1.44 (1.06-1.94) 0.99 (0.96-1.03) 0.99 (0.99-1)	0.018 0.804 0.371
Globulin GGT Bile.acid	0.022 0.006 0.005	2.02 6.5 2.68	1.02 (1-1.04) 1.01 (1-1.01) 1.00 (1-1.01)	0.043 <0.001 0.007

BCLC, Barcelona Clinic Liver Cancer; T.S, tumor size; Palb, prealbumin; GGT, gamma glutamyl transferase.

#### 3.2.2 Develop the nomogram

**Figure 4:**
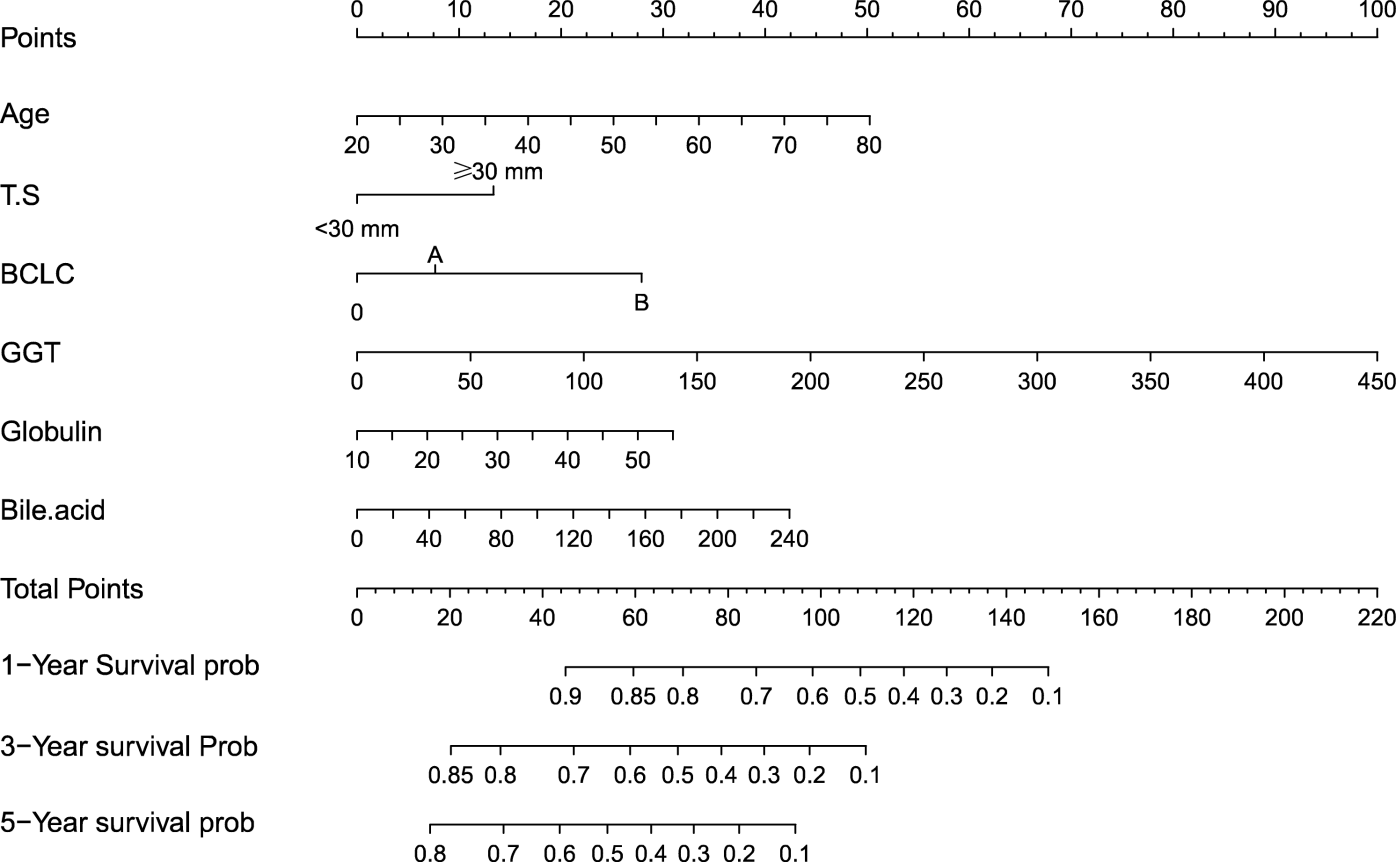
Nomogram, including Age, tumor number, BCLC stage, Globulin, GGT, and Bile acid for 1-, 3-, and 5- years recurrence free survival (RFS) in HCC patients with high HBsAg levels in AFP. The nomogram is valued to obtain the probability of 1-, 3-, and 5- years recurrence by adding up the points identified on the points scale for each variable. T.S, tumor size; BCLC, Barcelona Clinic Liver Cancer; GGT, gamma glutamyl transferase.

**Figure 5:**
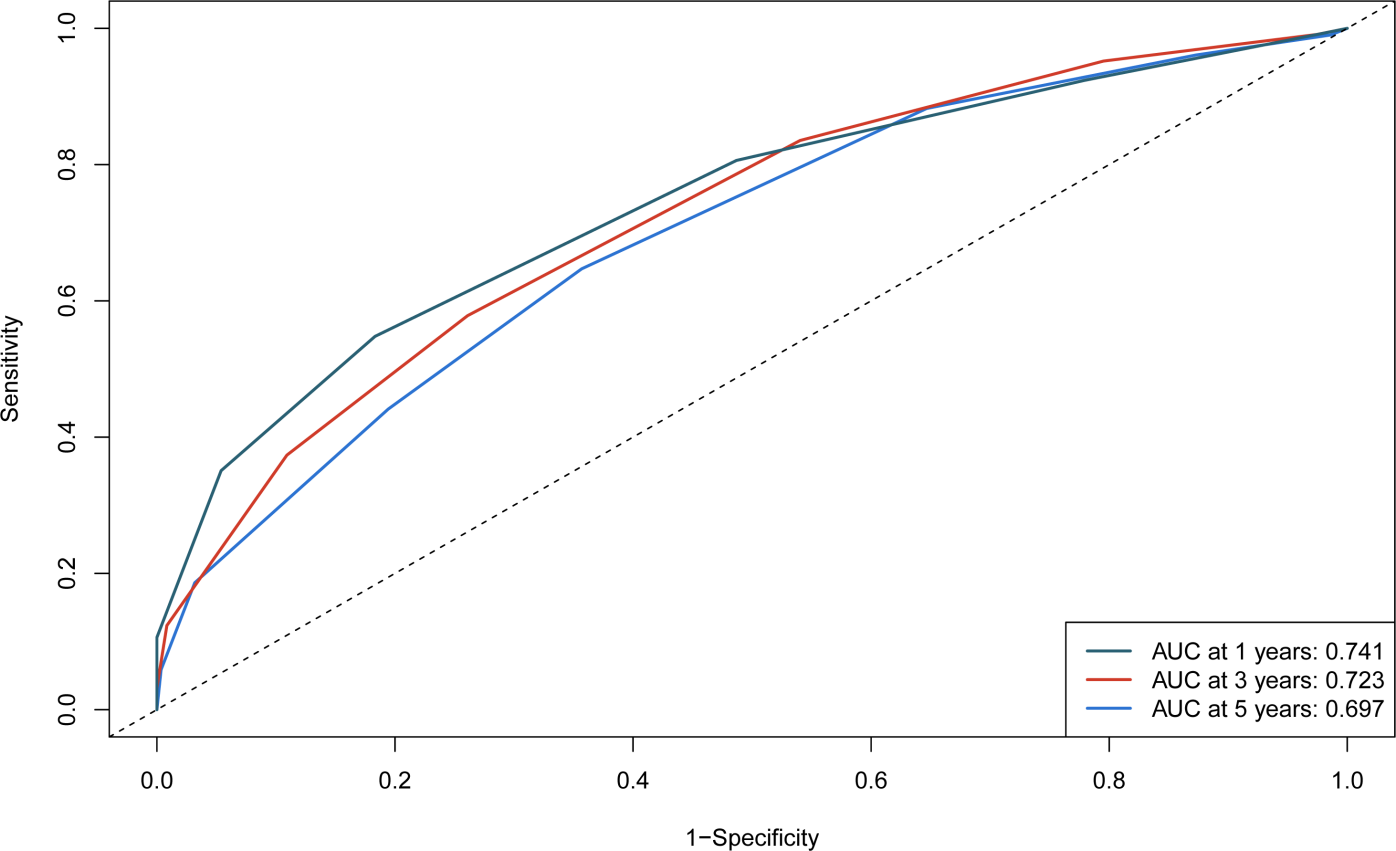
1-, 3-, and 5-year ROC curves of the nomogram in the training cohort.

**Figure 6:**
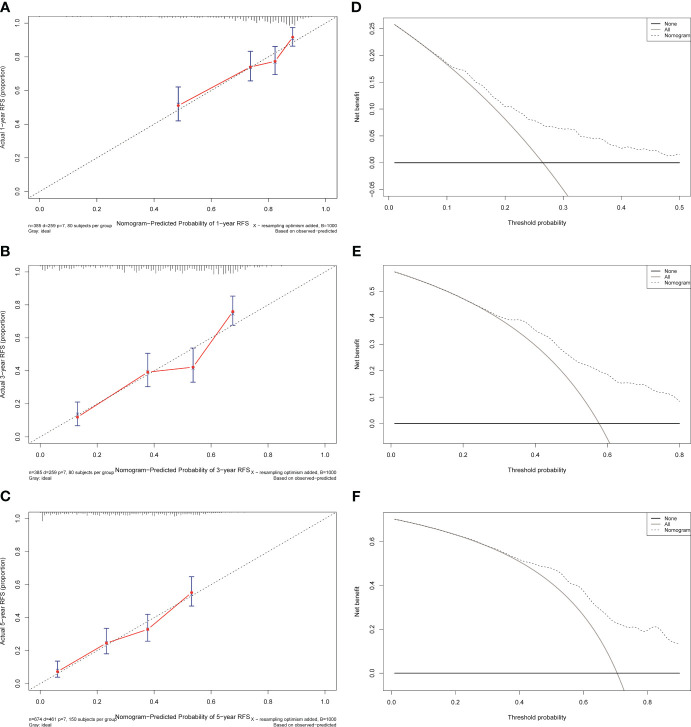
Calibration curves and decision curves analysis for recurrence of the nomogram in the training cohort. **(A)** One-year calibration curve in the training cohort. **(B)** Three-year calibration curve in the training cohort. **(C)** Five-year calibration curve in the training cohort. **(D)** One-year decision curve analysis in the training cohort. **(E)** Three-year decision curve analysis in the training cohort. **(F)** Five-year decision curve analysis in the training cohort.

The independent predictors found by the Lasso-Cox regression analysis were used to construct a nomogram (**Figure 4**). In the training cohort, the C-index was 0.682(95%CI: 0.639-0.725), and the time-dependent ROC curve demonstrated that AUCs of 1-, 3-, and 5-year were 0.741, 0.723, and 0.687 (**Figure 5**). It indicated the good predicting ability of our nomogram. The calibration curves of 1-, 3-, and 5-year demonstrated satisfactory accordance between the nomogram prediction and actual observation. In addition, the clinical value of the nomogram was evaluated using DCA, which provided the net benefits in reasonable threshold probability (**Figure 6**).

**Figure 7:**
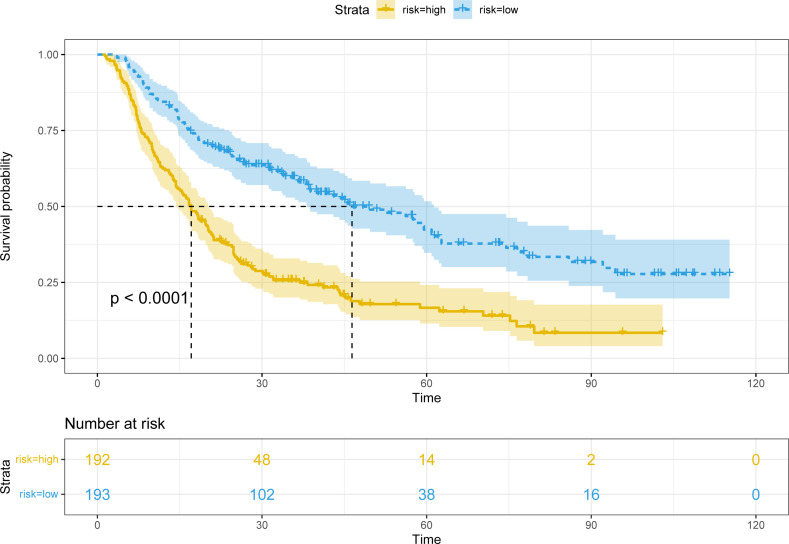
Kaplan-Meier plots of RFS for the low-risk group and high-risk group in the training cohort.

Patients were classified into two groups according to the score of the nomogram: low-risk group and high-risk group. In the training cohort, there were apparent variances in RFS (**Figure 7**) between the low-risk group (N=193) and high-risk group (N=192) (P<0.001).

#### 3.2.3 Validate the nomogram

To further test the efficacy of the reliability and robustness of our prognostic nomogram, internal and external validations were conducted on the nomogram. In the internal and external validation cohorts, the C-indexes of the nomogram for predicting the RFS were 0.666 (95%CI: 0.613-0.719) and 0.74 (95%CI: 0.696-0.783). The time-dependent ROC revealed that the AUCs of 1-, 3-, and 5-year were 0.702, 0.704, 0.684, 0.792, 0.734, and 0.770 in the internal and external validation cohorts (**Supplementary Figure S1**). The calibration curves also matched well (**Supplementary Figure S2**), and the DCA curves of 1-, 3-, and 5-year had good clinical practicability (**Supplementary Figure S3**).

The patients in two validation cohorts were also divided into high-risk and low-risk groups. The recurrence rates in the high-risk groups were significantly higher in the low-risk groups (P<0.001) (**Supplementary Figure S4**).

## 4 Discussion

HCC is one of the most common malignant tumors in the world. In China, the major etiology of the HCC is the HBV infection, which can promote the development and metastasis of the HCC (10, 24, 25). With the use of 1:1 PSM, our study found that the high level of HBsAg had a higher risk of recurrence than the low level of HBsAg. Consequently, our study is the first to focus on the high level of HBsAg patients who underwent TACE combined ablation to develop and validate a nomogram, which will hopefully predict the recurrence in H-HBsAg patients (High level of HBsAg). At present, there is a lack of a recurrence prediction model for H-HBsAg. We simultaneously created a nomogram by Lasso-Cox regression to accurately predict the prognosis of H-HBsAg patients.

The nomogram contains seven factors to produce the probability of an individual-specific clinical event, including age, tumor size, BCLC stage, globulin, GGT, and bile acid. The scores of the nomogram were obtained by drawing a vertical line at the location of the corresponding total score so that it intersected the three lines predicting the risk of recurrence, and the values shown at the intersection were predicted RFS at 1, 3, and 5 years. The C-index and AUCs of the training cohort and validation cohorts were similar, demonstrating adequate discrimination ability. The calibration curves presented the good prediction performance of the nomogram. Moreover, the nomogram indicated reliable clinical applicability by DCA curves. Patients were divided into two different risk groups according to the nomogram, and RFS was clearly different(P<0.001), which illustrated that our nomogram had a better ability to distinguish H-HBsAg patients to determine the risk of relapse after ablation therapy.

The number and size of tumors suggested strong tumor aggressiveness and poor prognosis of HCC, which was currently uncontroversial and needed not to be described here. Liver weight and portal blood flow velocity are reduced in the elderly, resulting in less reparability of the young body. Elderly people have lower immunity and faster tumor progression after treatment, leading to higher recurrence rates and worse prognosis (26, 27). At present, the BCLC system is regarded as an optimal staging system for tumor stage, treatment regimens, and expected survival. The expected survival rate is 50-70% for patients who are BCLC A at 5 years (28, 29). When we combined BCLC with other independent prognostic factors, the predictive value for prognosis could improve. GGT may be involved in the balance of oxidant and anti-oxidation, leading to sustained oxidative stress in tumor cells, which can contribute to the process of cancer (30, 31). Various proinflammatory proteins, including immunoglobulins, C-reactive protein, α2 macroglobulin, and fibrinogen are globulins (32, 33). Since human immunoglobulins are mainly metabolized by the liver, patients with severe hepatic dysfunction have a reduced ability to clear immunoglobulins, causing hyperglobulinemia (34, 35). Bile acid synthesis occurs in liver cells and is the end product of cholesterol metabolism (36). The Systemic homeostasis of bile acid mainly depends on its enterohepatic circulation process, which is of great significance for nutrient absorption and distribution, metabolic regulation, and homeostasis (37). Bile acid metabolism is implicated in tumor progression and hydrophobic bile acids are promoters of HCC (38, 39). Besides, reduced Farnesoid X (FXR) receptor signaling during hepatic inflammation induces to decrease in bile acid transporter proteins, resulting in elevated bile acids and persistent hepatic inflammation, which promote the development of HCC (40, 41).

The presence of HBsAg is a serologic marker of HBV infection and is used in clinical diagnosis (42, 43). HBsAg appears 1-2 weeks after exposure to HBV and precedes the onset of clinical symptoms and other serologic biochemical indicators of infection. There are still 257 million carriers of HBsAg despite the availability of antiviral therapeutics (44, 45). Many studies showed that the spontaneous HBsAg seroconversion rate was 1% and the presence of persistent HBsAg was associated with a high risk of HCC and a worse prognosis (46, 47). Previous studies by our team have also reported that the prognosis of HCC patients with negative HBsAg expression was better than that with positive HBsAg expression (48). In our study, we investigated the role of HBsAg levels in the recurrence of HCC after local treatment and used PSM to reduce bias. The results revealed that HBV-HCC patients with high HBsAg levels have worse prognosis than those with low HBsAg levels.

In the BCLC Guideline, TACE is recommended for BCLC intermediate stage B HCC. For early-stage HCC, TACE can mark the tumor and achieve tumor downstaging, thereby declining the time and increasing the success rate of ablation (49). Foreign and domestic studies have suggested that combination therapy by TACE and ablation improved overall and progression-free survival compared with TACE alone (50, 51). Unlike the conventional univariate analysis, the LASSO regression that we used aimed to select variables for Cox regression to avoid overfitting. Also, the nomogram can be validated by both internal and external validation because our study was a multicenter retrospective study. Simultaneous examination of comprehensive patient features covering demographics, liver function, tumor load, tumor markers, and inflammatory markers was a major strength of our study. The consists of our nomogram are simple and easy to obtain so that the clinicians are able to evaluate the patient’s condition in a timely and effective manner.

Several limitations of our study should be addressed. The first one of them is the retrospective nature and it is necessary to strengthen the conclusions by further validations in large prospective studies. Because as a retrospective study, there is inevitable selection bias. Although internal and external validations were conducted by a larger multicenter sample, external validations from other centers are still required in the future. Besides, the patients included in our study all received TACE combined with ablation. Whether the nomogram would be suitable for other treatments such as surgery and liver transplantation requires further investigation. Lastly, the study was conducted only in China, where hepatitis B virus is the principal cause of HCC. Thus, generalizing to other populations in which HBV is not a major causative factor for HCC must be carried out with caution. Nevertheless, we used up to eight years of follow-up to create an accurate and reliable nomogram to better guide clinical practice for this group of HCC patients with high levels of HBsAg. In general, high-risk patients needed more frequent clinical surveillance and appropriate interventions to prevent recurrence and progression.

## 5 Conclusion

In summary, high levels of HBsAg were associated with tumor progression and poor prognosis. For high levels of HBsAg patients, we created an accurate and reliable nomogram to predict recurrence based on the Lasso-Cox regression analysis. The nomogram, including age, BCLC stage, tumor size, globulin, GGT, and bile acids, demonstrated adequate discrimination ability, which could better guide the clinical decisions.

## Data availability statement

The raw data supporting the conclusions of this article will be made available by the authors, without undue reservation.

## Ethics statement

Ethical approval was not required for the study involving humans in accordance with the local legislation and institutional requirements. Written informed consent to participate in this study was not required from the participants or the participants’ legal guardians/next of kin in accordance with the national legislation and the institutional requirements.

## Author contributions

YX: Data curation, Writing – original draft, Writing – review & editing. WQ: Validation, Visualization, Writing – review & editing. QW: Data curation, Writing – review & editing. KL: Supervision, Writing – review & editing. RJ: Resources, Supervision, Writing – review & editing. YZ: Resources, Writing – review & editing.

## Supplementary material

Supplementary Figure 1 A. 1-, 3-, and 5-year ROC curves of the nomogram in the internal validation cohort. B. 1-, 3-, and 5-year ROC curves of the nomogram in the external validation cohort.

Supplementary Figure 2 Calibration curves for recurrence of the nomogram in the validation cohorts. **(A)** One-year calibration curve in the internal validation cohort. **(B)** Three-year calibration curve in the internal validation cohort. **(C)** Five-year calibration curve in the internal validation cohort. **(D)** One-year calibration curve in the external validation cohort. **(E)** Three-year calibration curve in the external validation cohort. **(F)** Five-year calibration curve in the external validation cohort.

Supplementary Figure 3 Decision curves analysis for recurrence in the internal validation cohort. **(A)** Decision curve analysis for one-year RFS in the internal validation cohort. **(B)** Decision curve analysis for three-year RFS in the internal validation cohort. **(C)** Decision curve analysis for five- year RFS in the internal validation cohort. **(D)** Decision curve analysis for one-year RFS in the external validation cohort. **(E)** Decision curve analysis for three-year RFS in the external validation cohort. **(F)** Decision curve analysis for five- year RFS in the external validation cohort.

Supplementary Figure 4
**(A)** Kaplan-Meier plots of RFS for the low-risk group and high-risk group in the internal validation cohort. **(B)** Kaplan-Meier plots of RFS for the low-risk group and high-risk group in the external validation cohort.
