# Supplementary figures and images for: Construction and validation of a machine learning-based nomogram to predict the prognosis of HBV associated hepatocellular carcinoma patients with high levels of hepatitis B surface antigen in primary local treatment: a multicenter study
Source: Front Immunol. 2024 Mar 27;15:1357496. doi: 10.3389/fimmu.2024.1357496 (PMC11004323; doi:10.3389/fimmu.2024.1357496)

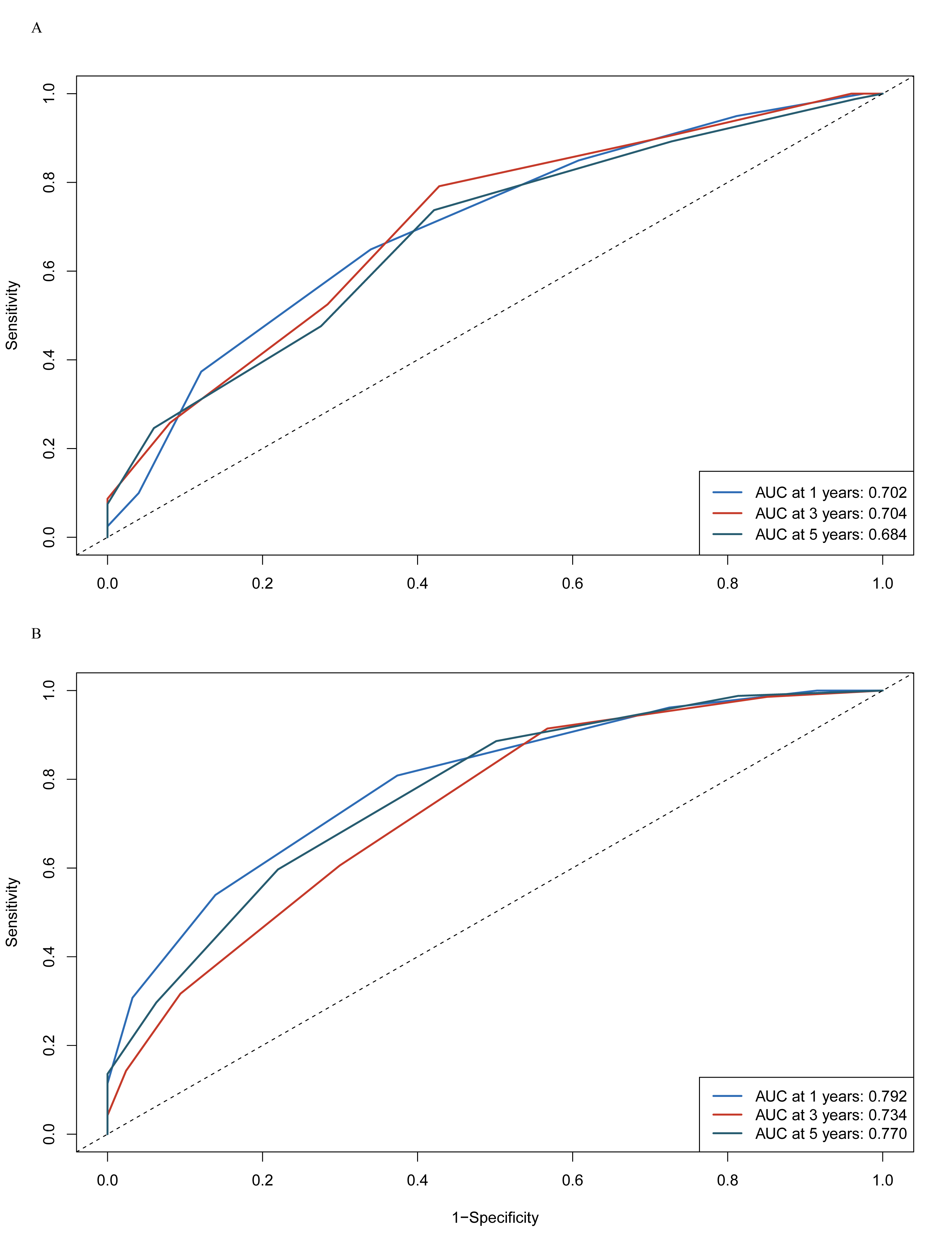

Supplement: Supplementary Figure 1 — A. 1-, 3-, and 5-year ROC curves of the nomogram in the internal validation cohort. B. 1-, 3-, and 5-year ROC curves of the nomogram in the external validation cohort. [file Image_1.tif]

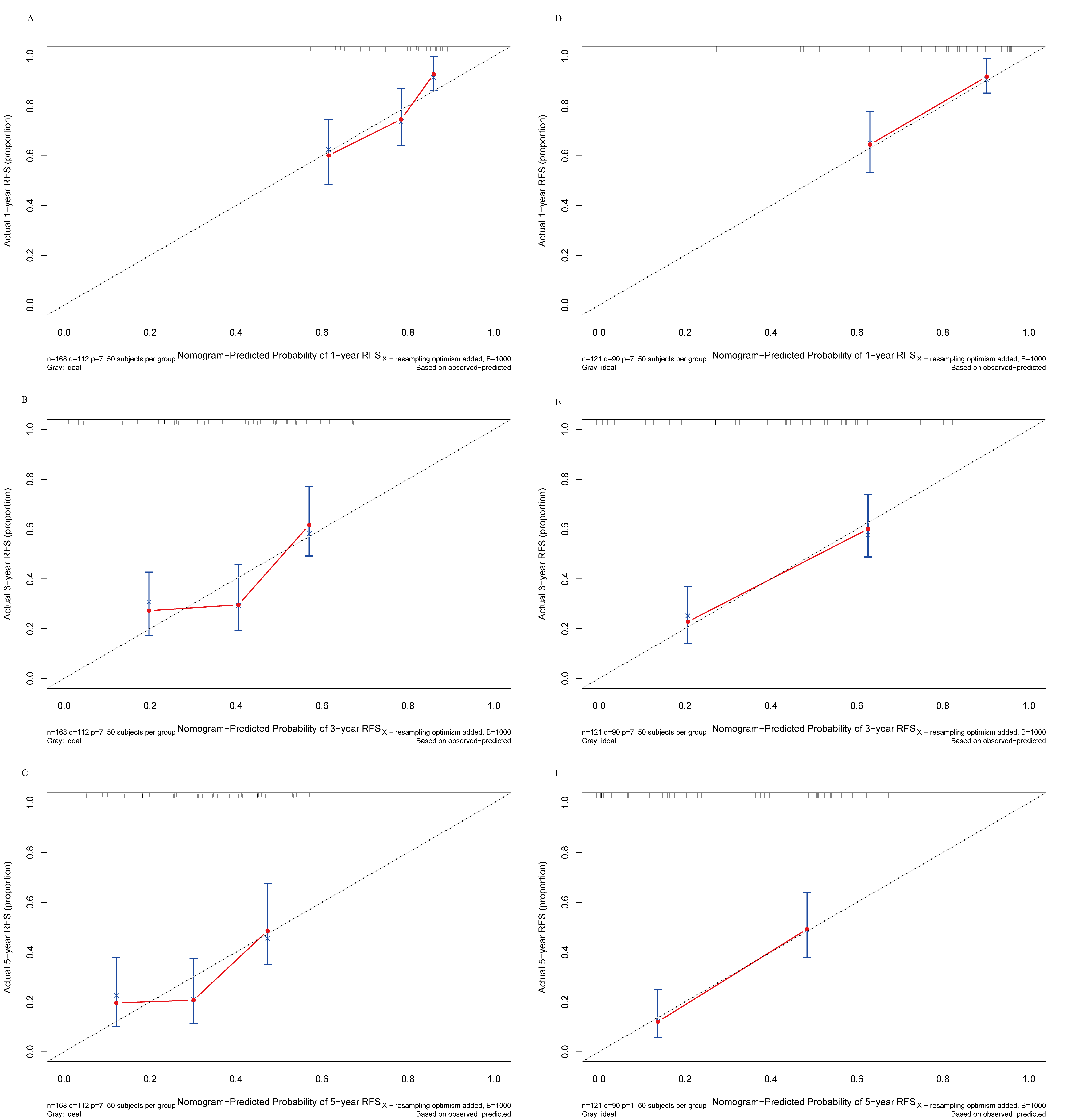

Supplement: Supplementary Figure 2 — Calibration curves for recurrence of the nomogram in the validation cohorts. (A) One-year calibration curve in the internal validation cohort. (B) Three-year calibration curve in the internal validation cohort. (C) Five-year calibration curve in the internal validation cohort. (D) One-year calibration curve in the external validation cohort. (E) Three-year calibration curve in the external validation cohort. (F) Five-year calibration curve in the external validation cohort. [file Image_2.tif]

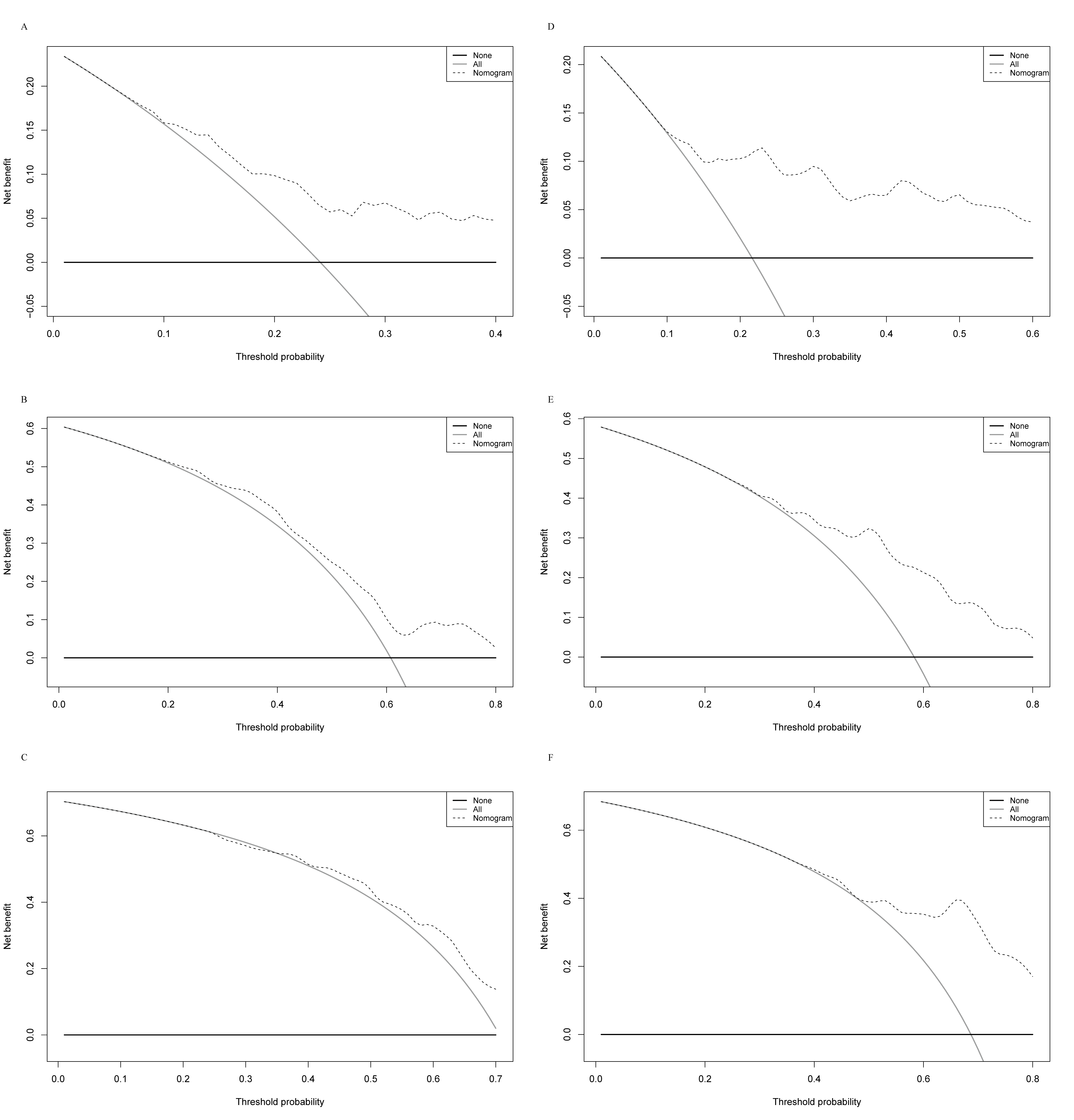

Supplement: Supplementary Figure 3 — Decision curves analysis for recurrence in the internal validation cohort. (A) Decision curve analysis for one-year RFS in the internal validation cohort. (B) Decision curve analysis for three-year RFS in the internal validation cohort. (C) Decision curve analysis for five- year RFS in the internal validation cohort. (D) Decision curve analysis for one-year RFS in the external validation cohort. (E) Decision curve analysis for three-year RFS in the external validation cohort. (F) Decision curve analysis for five- year RFS in the external validation cohort. [file Image_3.tif]

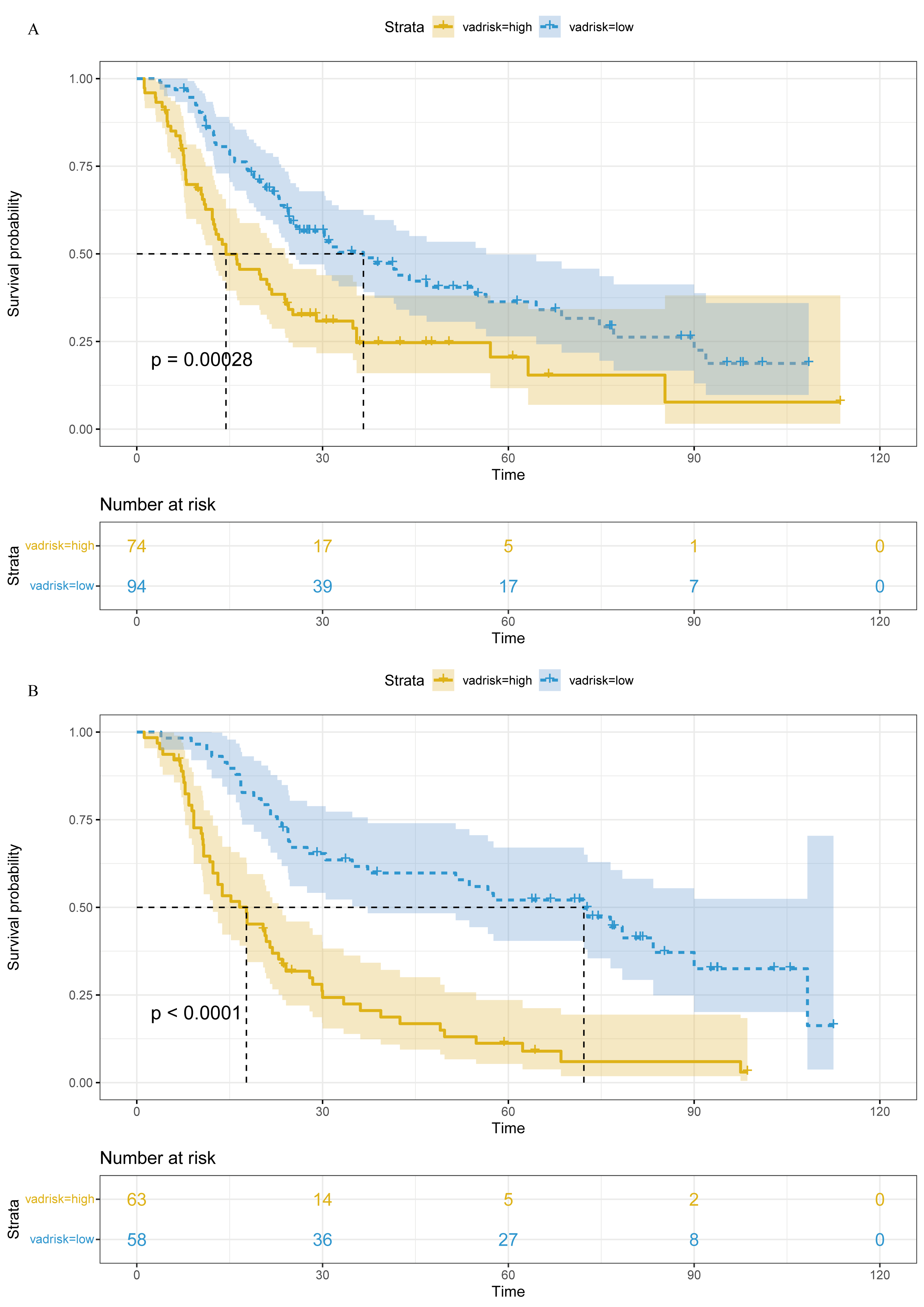

Supplement: Supplementary Figure 4 — (A) Kaplan-Meier plots of RFS for the low-risk group and high-risk group in the internal validation cohort. (B) Kaplan-Meier plots of RFS for the low-risk group and high-risk group in the external validation cohort. [file Image_4.tif]
